# S1-Leitlinie: Tumorgenetik – Diagnostik im Kontext maligner Erkrankungen

**DOI:** 10.1515/medgen-2022-2112

**Published:** 2022-05-07

## Federführende Fachgesellschaften

Deutsche Gesellschaft für Humangenetik (GfH)

Deutsche Gesellschaft für Pathologie (DGP)

## Expertengruppe

**Table:** 

Stefan Aretz	Institut für Humangenetik, Universitätsklinikum Bonn
Bernd Auber	Institut für Humangenetik, Medizinische Hochschule Hannover
Daniela Aust	Institut für Pathologie, Universitätsklinikum Dresden
Gustavo Baretton	Institut für Pathologie, Universitätsklinikum Dresden
Falko Fend	Institut für Pathologie und Neuropathologie, Universitätsklinikum Tübingen
Laura Gieldon	Institut für Humangenetik, Universitätsklinikum Heidelberg
Gudrun Göhring	Institut für Humangenetik, Medizinische Hochschule Hannover
Lana Harder	Institut für Tumorgenetik Nord, Kiel
Arndt Hartmann	Pathologisches Institut, Universitätsklinikum Erlangen
Elke Holinski-Feder	Medizinisch Genetisches Zentrum, München
Andreas Laner	Medizinisch Genetisches Zentrum, München
Silke Laßmann	Institut für Klinische Pathologie, Universitätsklinikum Freiburg
Sabine Merkelbach-Bruse	Institut für allgemeine Pathologie und Anatomie, Universitätsklinikum Köln
Brigitte Schlegelberger	Institut für Humangenetik, Medizinische Hochschule Hannover
Evelin Schröck	Institut für klinische Genetik, Technische Universität Dresden
Christopher Schroeder	Institut für Medizinische Genetik und Angewandte Genomik, Universitätsklinikum Tübingen
Felix Sahm	Institut für Pathologie, Abteilung für Neuropathologie, Universitätsklinikum Heidelberg
Peter Schirmacher	Institut für Pathologie, Universitätsklinikum Heidelberg
Reiner Siebert	Institut für Humangenetik, Universitätsklinikum Ulm
Albrecht Stenzinger	Institut für Pathologie, Universitätsklinikum Heidelberg
Markus Tiemann	Institut für Hämatopathologie, Hamburg
Sebastian Wagner	Medizinische Klinik 2, Universitätsklinikum Frankfurt
Wilko Weichert	Institut für Pathologie, Technische Universität München

## Von den Fachgesellschaften/Verbänden benannte Mandatsträgerinnen und Mandatsträger

**Table:** 

Nils Rahner	(Stellvertreterin: Verena Steinke-Lange; Berufsverband Deutscher Humangenetiker)
Karl-Friedrich Bürrig	(Bundesverband Deutscher Pathologen)
Tanja Fehm	(Deutsche Gesellschaft für Gynäkologie und Geburtshilfe)
Sebastian Wagner	(Deutsche Gesellschaft für Hämatologie und Onkologie)
Hanns-Georg Klein	(Deutsche Gesellschaft für Klinische Chemie)
Felix Sahm	(Deutsche Gesellschaft für Neuropathologie und Neuroanatomie)
Arndt Borkhardt	(Stellvertreter: Markus Metzler; Gesellschaft für Pädiatrische Onkologie und Hämatologie)
Rita Schmutzler	(Arbeitskreis erbliche Tumorerkrankungen (AET) der Deutschen Krebsgesellschaft)

## Diese S1 Leitlinie wird durch folgende Fachgesellschaften/Berufsverbände mitgetragen:

Berufsverband Deutscher Humangenetiker (BVDH)

Bundesverband Deutscher Pathologen (BDP)

Deutsche Gesellschaft für Gynäkologie und Geburtshilfe e.V. (DGGG)

Deutsche Gesellschaft für Hämatologie und Onkologie (DGHO)

Deutsche Gesellschaft für Klinische Chemie (DGKL)

Deutsche Gesellschaft für Neuropathologie und Neuroanatomie (DGNN)

Gesellschaft für Pädiatrische Onkologie und Hämatologie (GPOH)

Arbeitskreis erbliche Tumorerkrankungen (AET) der Deutschen Krebsgesellschaft (DKG)

## Inhaltsverzeichnis

**FEDERFÜHRENDE FACHGESELLSCHAFTEN**

Expertengruppe

Von den Fachgesellschaften/Verbänden benannte Mandatsträgerinnen und Mandatsträger

Diese S1 Leitlinie wird durch folgende Fachgesellschaften/Berufsverbände mitgetragen

**PRÄAMBEL**

**SPEZIFISCHE STATEMENTS**

1. Statement: Bei der Indikationsstellung zur tumorgenetischen Diagnostik soll geklärt werden, ob ausschließlich hinsichtlich diagnostisch oder therapeutisch relevanter somatischer Varianten oder ob (auch) auf diagnostisch oder therapeutisch relevante pathogene Keimbahnvarianten untersucht werden soll.
2. Statement: Die diagnostische und therapiesteuernde molekulare Diagnostik bei soliden Tumoren sollte in die gesamte gewebebasierte Diagnostik sowie die interdisziplinäre Indikationsstellung und Therapieentscheidung eingebettet sein.
3. Statement: Vor einer tumorgenetischen Diagnostik soll eine dem diagnostischen Umfang angepasste qualifizierte Aufklärung durchgeführt werden.
4. Statement: Die Untersuchung auf Keimbahnvarianten erfordert eine Aufklärung nach Gendiagnostikgesetz.
5. Eine humangenetische Beratung sollte empfohlen werden, wenn klinische oder familienanamnestische Hinweise auf ein Tumorrisikosyndrom (TRS) bestehen.
6. Statement: Eine Analyse von Nur-Tumor-Material kann eine erbliche Tumordisposition weder nachweisen noch ausschließen. Dies ist bei der Indikationsstellung zu berücksichtigen.
7. Statement: Bei Verdacht auf ein erbliches TRS sollte immer eine genetische Untersuchung auf Keimbahnebene angestrebt werden, die alle mit dem TRS assoziierten Gene beinhaltet
8. Statement: Die Aussagekraft der tumorgenetischen Diagnostik ist abhängig vom verwendeten Ausgangsmaterial – dies sollte durch entsprechende Angaben im Befundtext nachvollziehbar gemacht werden.
9. Bei der Untersuchung von Tumor- und nicht-Tumorproben sollte die Herkunft und Qualität der Proben sachgerecht validiert werden.
10. Statement: Die tumorgenetische Diagnostik soll von entsprechenden qualitätssichernden Maßnahmen begleitet werden.
11. Statement: Bei der tumorgenetischen Diagnostik sollte berücksichtigt werden, dass auch Varianten im Mosaikstatus nachgewiesen werden können, welche auch in anderen, nicht-tumortragenden Geweben, z. B. in den Keimzellen, vorliegen können.
12. Statement: Bei Verwendung von Blut oder Speichel als nicht-Tumorprobe sollten mögliche klonale Ereignisse berücksichtigt werden.
13. Statement: Bei hämatologischen Neoplasien sind bei der Untersuchung auf Keimbahnvarianten besondere Vorkehrungen bei der Auswahl des untersuchten Gewebes zu treffen.
14. Statement: Um therapeutisch relevante Varianten zu erkennen, kann die Liquid Biopsy in definierten klinischen Konstellationen als komplementäre Methode zur gewebebasierten Analytik genutzt werden.
15. Statement: Die Liquid Biopsy wird stark durch biologisch-klinische, präanalytische und analytische Faktoren beeinflusst, die kontrolliert und bei der Analyse und Befundinterpretation beachtet werden müssen.
16. Statement: Vor dem Einsatz einer ctDNA-Analyse zur Detektion von Varianten, die als prädiktive Biomarker oder therapeutische Zielstrukturen genutzt werden können, sollte die histopathologische oder hämatologische Diagnose stehen.
17. Statement: Für eine eindeutige Zuordnung von konstitutionellen und somatischen Varianten bei einer tumorgenetischen Diagnostik von zirkulierender zellfreier DNA („Liquid Biopsy“) ist eine parallele Analyse von nicht-Tumor-DNA erforderlich.
18. Statement: Die Nennung definierter präanalytischer und analytischer Testparameter soll Bestandteil des diagnostischen Befundberichtes sein.
19. Statement: Genetische Varianten sollen nach definierten Nomenklatur-Standards beschrieben werden.
20. Statement: Bei paralleler Untersuchung an Tumor- und Normalproben muss sichergestellt werden, dass relevante (potentiell) erbliche Varianten nicht pauschal „maskiert“ werden.
21. Statement: Die Begriffe „Mutation“ bzw. „Polymorphismus“ sollen nicht zur Beschreibung von Varianten in tumorgenetischen Befunden verwendet werden.
22. Statement: In der tumorgenetischen Diagnostik soll die biologische und klinische Bewertung genetischer Varianten mittels eines konsentierten Standards erfolgen.
23. Statement: Bei allen umfangreichen tumorgenetischen Untersuchungen an Tumormaterial sollten potentiell erbliche Varianten, die mit einem TRS assoziiert sein könnten, berichtet werden.
24. Statement: Die Ergebnisse von Variantenbewertungen sollten in öffentlichen Datenbanken abgelegt werden.
25. Statement: Bei umfangreichen tumorgenetischen Untersuchungen ist eine interdisziplinäre Bewertung der Ergebnisse anzustreben.

**QUELLEN/LITERATURANGABEN**

**APPENDIX**

## Präambel

Die im Rahmen von Tumorerkrankungen in der Humangenetik und der Pathologie durchgeführte molekulargenetische Diagnostik ist eine wesentliche Voraussetzung für die moderne, individualisierte Präzisionsmedizin und gewinnt daher für die Tumortherapie und -prävention zunehmend an Bedeutung. Entsprechende Untersuchungen werden immer umfassender eingesetzt und reichen von der gezielten Analyse einzelner, klar umschriebener Regionen hin zu weit gefassten genetischen Analysen mit Hilfe sogenannter Genpanels oder Ganzexom- (WES) oder Ganzgenomanalysen (WGS). Die diagnostische Analyse mittels großer Genpanels, des Exoms oder des Genoms wird in den kommenden Jahren eine zunehmende Rolle spielen.

Die Analysen, die in der Humangenetik und Molekularpathologie durchgeführt werden, sollen in einem qualitätsgesicherten Umfeld erfolgen und umfassen einen diagnostischen Gesamtprozess, der von der Präanalytik des Probenmaterials über die Sequenzierung bis zur Identifizierung, Annotation und Klassifikation sowie klinischen Interpretation von Varianten reicht. Relevant ist die Einbettung der humangenetischen und molekularpathologischen Analytik in einen klinischen Kontext, der u. a. die Indikationsstellung, Beratungsleistungen und ggf. Beratungen durch das interdisziplinäre Organboard oder das molekulare Tumorboard umfasst.

Durch den Einsatz einer umfangreichen Analytik primär an Tumor-DNA (sog. ‚tumor-only‘ Untersuchungen) zur Identifizierung von somatischen therapeutischen Zielstrukturen sowie diagnostischen und prädiktiven Biomarkern (einschließlich Resistenzmechanismen, die das Therapieansprechen vorhersagen), steigt auch die Wahrscheinlichkeit, Varianten zu detektieren, die möglicherweise nicht auf die Krebszellen begrenzt sind, sondern potentiell erbliche (konstitutionelle) Varianten darstellen. Auch in der WES- und WGS- Analytik von Tumoren, die in der Regel unter Einschluss einer parallelen Analyse konstitutioneller Varianten (Keimbahnanalyse) durchgeführt wird, spielt neben der Detektion somatischer Varianten die Erkennung konstitutioneller Varianten eine wichtige Rolle.

Je nach Tumorart und betroffenem Gen, insbesondere bei Tumorsuppressor- und DNA-Reparaturgenen, kann bei der Analyse von Tumormaterial die Wahrscheinlichkeit des Vorliegens einer konstitutionellen Variante sogar höher als die einer somatischen Veränderung sein (z. B. der Nachweis einer *BRCA1*-Variante im Ovarialkarzinom) [1], [2]. Eine weitere Charakterisierung solcher Varianten hinsichtlich potentieller Erblichkeit ist je nach tumorgenetischem Befund, Fragestellung und klinischem Kontext wichtig für die korrekte Einschätzung der klinischen Bedeutung, sowohl für die untersuchte Person selber als auch für deren Familie. Für die Indikation bei manchen zielgerichteten Therapien, z. B. der PARP-Inhibitor-Gabe im Rahmen einer Erhaltungstherapie beim metastasierten Adenokarzinom des Pankreas, ist der Nachweis einer *BRCA1*- oder *BRCA2*-Keimbahnvariante zurzeit sogar zwingend erforderlich.

Darüber hinaus ist die Unterscheidung zwischen einer somatischen oder konstitutionellen pathogenen *BRCA1*-Variante nicht nur für die untersuchte Patientin z. B. bzgl. ihres deutlich erhöhten Brustkrebsrisikos, sondern auch für weitere (asymptomatische) Familienangehörige medizinisch relevant. Sie können nach humangenetischer Beratung und Einwilligung nach dem Gendiagnostikgesetz (GenDG) eine prädiktive (vorhersagende) Testung durchführen lassen, um zu klären, ob sie die pathogene Keimbahnvariante geerbt haben und ein erhöhte Krebsrisiko tragen oder nicht. Ebenso ist im Umkehrschluss die korrekte Einordnung einer Variante als „somatisch“ essentiell, um die fehlerhafte Diagnose eines erblichen Tumorrisikosyndroms zu vermeiden. Deswegen ist bei umfangreichen Untersuchungen eine parallele Sequenzierung von DNA aus Tumormaterial und peripherem Blut anzustreben [3]. Alleine durch klinische Informationen oder die Familienanamnese ist nur unzureichend feststellbar, ob eine Person möglicherweise eine Tumordisposition trägt. Solche erblichen Tumorrisikosyndrome sind auch bei pauschal als „sporadisch“, d. h. als Einzelfall in der Familie eingeschätzten Krebserkrankungen deutlich häufiger als bisher angenommen [4], [5], [6], [7] und werden bei mindestens 10 % aller umfangreichen tumorgenetischen Untersuchungen gefunden [3], [8], [9], [10].

Das Spektrum an genetischen Veränderungen in der tumorgenetischen Diagnostik ist sehr groß und umfasst von Einzelbasenveränderungen bis zu komplexen strukturellen Varianten (wie bspw. Kopienzahlveränderungen und *in-frame* Genfusionen), Signaturen und komplexe Biomarker (bspw. Tumormutationslast (TMB) und genomische Instabilität durch homologe Reparaturdefizienz (HRD)) ein wachsendes Feld an Alterationen, das eine spezifische Expertise erfordert. Dies schließt auch eine dezidierte Kenntnis der klinischen Studienlandschaft und der aktuellen Zulassungssituationen ein.

Die Durchführung sowohl einer gezielten als auch einer umfangreichen genetischen Diagnostik im Kontext maligner Erkrankungen und die Variantenbeschreibung und -bewertung sollte daher interdisziplinär, evidenzbasiert und standardisiert erfolgen, um einen maximalen Nutzen für die Patienten zu erzielen.

Diese Leitlinie soll aufzeigen, welche Aspekte diesbezüglich zu beachten sind.

## Spezifische Statements

### 1. Statement: Bei der Indikationsstellung zur tumorgenetischen Diagnostik soll geklärt werden, ob ausschließlich hinsichtlich diagnostisch oder therapeutisch relevanter somatischer Varianten oder ob (auch) auf diagnostisch oder therapeutisch relevante pathogene Keimbahnvarianten untersucht werden soll

**Kommentar:** Ziel der tumorgenetischen Diagnostik bei malignen Erkrankungen ist es, genetische Varianten zu detektieren, die eine diagnostische, prognostische oder therapeutische Relevanz haben im Sinne einer prädiktiven Biomarkeranalyse zur Therapiesteuerung. Somatische Varianten werden in der Regel durch die Analyse von Tumor-DNA nachgewiesen, wobei naturgemäß auch Keimbahnvarianten miterfasst werden. Hingegen werden konstitutionelle Varianten („Keimbahnvarianten“) in der Regel durch die Untersuchung von Leukozyten-DNA aus venösem Blut nachgewiesen, seltener und je nach Fragestellung (z. B. bei hämatologischen Neoplasien) auch aus anderem gesunden Gewebe.

**Abb. 1:**
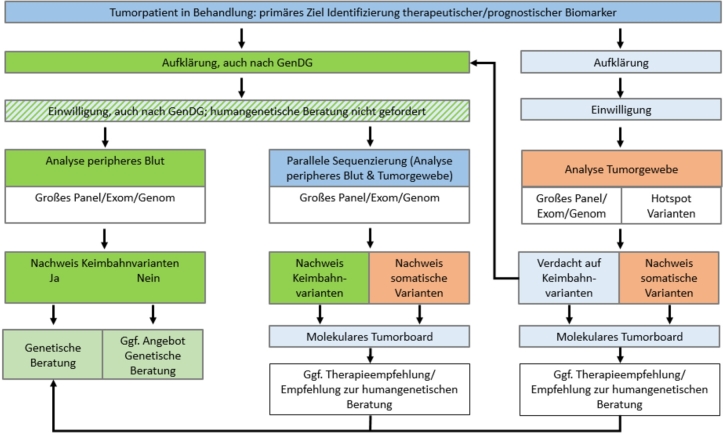
Patientenpfad Tumorgenetik. Je nach Intention der tumorgenetischen Diagnostik gibt es unterschiedliche Anforderungen an Aufklärung und Einwilligung des Patienten (GenDG: Gendiagnostikgesetz).

Sollen ausschließlich Keimbahnvarianten, die bei verschiedenen Tumoren ebenfalls prädiktive therapeutische Relevanz haben, untersucht werden, soll zunächst eine Keimbahnanalyse an Blut erfolgen, weil nur so das gesamte Spektrum möglicher Keimbahnvarianten sicher nachgewiesen werden kann. Da die Unterscheidung zwischen erblichen und somatischen Varianten je nach klinischer Konstellation auf Grund der unterschiedlichen klinisch-humangenetischen Implikationen eine hohe Relevanz aufweisen kann, ist bei umfangreichen Analysen und entsprechender klinischer Fragestellung eine parallele Sequenzierung von DNA aus Tumormaterial und peripherem Blut anzustreben. Abbildung 1 zeigt die derzeitigen klinischen Konstellationen auf, in denen die Keimbahn- und somatische Analyse eine Rolle spielt.

### 2. Statement: Die diagnostische und therapiesteuernde molekulare Diagnostik bei soliden Tumoren sollte in die gesamte gewebebasierte Diagnostik sowie die interdisziplinäre Indikationsstellung und Therapieentscheidung eingebettet sein

**Kommentar:** Die molekularpathologische Diagnostik bei soliden Tumoren ist immer in einen klinischen und histopathologischen Kontext eingebettet. Dieser Kontext schließt die fachgerechte Indikationsstellung und interdisziplinäre Therapieentscheidung auf der Basis eines diagnostischen Befundes ein. Darüber hinaus dient der klinische und histopathologische Kontext der Plausibilitätsprüfung des molekularpathologischen Diagnostikprozesses einschließlich des Ergebnisses und unterstützt somit die Qualität des molekularpathologischen Befundes. Die isolierte molekularpathologische Untersuchung von DNA oder RNA aus Tumorgewebe ohne Kenntnis der histopathologischen (und ggf. immunhistologischen) Diagnose (Tumortyp) und der verwendeten Präanalytik, einschließlich der Detektion und Einzeichnung des tumortragenden Areals im Gewebeschnitt für die Nukleinsäureextraktion und der verwendeten Extraktionsmethoden, kann zu gravierenden Fehlern in der molekularpathologischen Diagnostik führen (bspw. falsch-negative Befunde bei Isolation von DNA aus nicht-tumor tragenden Gewebesegmenten oder bei zu niedrigem Tumorzellgehalt) und soll daher nicht durchgeführt werden. Da verschiedene genetische Aberrationen in unterschiedlicher Prävalenz mit bestimmen Tumortypen vergesellschaftet sind, unterstützt die Korrelation zwischen dem histologischen Phänotyp und dem somatischen Genotyp des Tumors ebenfalls die diagnostische Plausibilitätsprüfung. Ein integrierter molekularpathologischer Befund, der klinische, histopathologische und molekularpathologische Ergebnisse kontextualisiert, ist daher ein wesentlicher Bestandteil der molekularpathologischen Analytik.

### 3. Statement: Vor einer tumorgenetischen Diagnostik soll eine dem diagnostischen Umfang angepasste qualifizierte Aufklärung durchgeführt werden

**Kommentar:** Je nach Umfang, Art und Intention der geplanten tumorgenetischen Diagnostik ist die Patientin/der Patient vor Durchführung über die Bedeutung und Tragweite der geplanten Untersuchung wirksam aufzuklären. Dabei sollte berücksichtigt werden, dass die Untersuchung – außer wenn im Tumorgewebe auf einige wenige, bekannte somatische pathogene Treiber-Varianten (bspw. fokussierte sog. Hotspot Panels) getestet wird – Hinweise auf ein erbliches Tumorrisikosyndrom ergeben kann, und dass sich daraus potentiell Konsequenzen nicht nur für die Betroffenen selbst, sondern auch ihre Angehörigen ergeben können. In solchen Fällen sollte entweder primär oder sekundär (siehe Abb. 1) eine Aufklärung nach dem GenDG erfolgen. Im Patientenrechtegesetz (§ 630 c und e) wird auf eine umfassende Aufklärungspflicht hingewiesen.

**Tab. 1:** Anforderungen an die Aufklärung vor tumorgenetischen Untersuchungen.

Untersuchungsziel (Intention)	Aufklärung gemäß Patientenrechtegesetz	Aufklärung gemäß Gendiagnostikgesetz
Gezielter Nachweis therapierelevanter **Varianten im Tumorgewebe**	ja	nein
Großes Panel/Exom/Genom an Tumormaterial, ggf. mit **Hinweis auf** potentielle **Keimbahnvariante**	ja	nein (ggf. sekundär, siehe Abb. 1)
**Diagnostische Aussage zu** therapierelevanten **Keimbahnvarianten**	ja	ja

### 4. Statement: Die Untersuchung auf Keimbahnvarianten erfordert eine Aufklärung nach Gendiagnostikgesetz

**Kommentar:** Das „Gesetz über genetische Untersuchungen bei Menschen“ (Gendiagnostikgesetz – GenDG) regelt die Voraussetzungen für genetische Untersuchungen von genetischen Eigenschaften. Als „genetische Eigenschaften“ werden laut GenDG „während der Befruchtung oder bis zur Geburt erworbene, vom Menschen stammende Erbinformationen“ bezeichnet. Ererbte oder während der Befruchtung erworbene genetische Veränderungen sind meist in allen Körperzellen nachweisbar und werden dann in aller Regel auch in der Analyse des Tumorgewebes detektiert. Bei einer umfangreichen genetischen Diagnostik an Tumormaterial kann somit auch eine relevante erbliche Variante nachgewiesen werden. Ob eine Aufklärung (und Einwilligung) nach GenDG vor einer tumorgenetischen Diagnostik erfolgen muss, richtet sich primär nach der Intention der geplanten Untersuchung. Ist es das vorrangige Ziel, in Tumor-DNA vorkommende „Biomarker“ zu identifizieren, fällt dies zunächst nicht in den Geltungsbereich des GenDG. Dennoch kann je nach Kontext entsprechend Patientenrechtegesetz eine umfassendere Aufklärung indiziert sein, s. dazu auch Statement 3 sowie den 2. bzw. 3. Tätigkeitsbericht der Gendiagnostikkommission (Tätigkeitsbericht für den Zeitraum 2013-2015, S. 51ff bzw. Tätigkeitsbericht für den Zeitraum 2016-2018, S. 15). Ist der Nachweis einer Keimbahnvariante die Grundlage einer zielgerichteten Therapie, ist eine Aufklärung nach GenDG zwingend vorgeschrieben. Eine Übersicht hinsichtlich der Anforderungen an die Aufklärung abhängig von den verschiedenen Untersuchungszielen ist in Tab. 1 dargestellt.

Eine zusätzliche präanalytische humangenetische Beratung nach GenDG ist in der tumorgenetischen Diagnostik, auch bei Durchführung einer Keimbahndiagnostik, meist nicht gefordert, da in aller Regel eine bereits erkrankte Person untersucht wird, d. h. eine diagnostische genetische Untersuchung erfolgt.

### 5. Statement: Eine humangenetische Beratung sollte empfohlen werden, wenn klinische oder familienanamnestische Hinweise auf ein Tumorrisikosyndrom (TRS) bestehen

**Kommentar:** Hierzu zählt z. B. das Auftreten weiterer Tumorerkrankungen bei der erkrankten Person selbst oder in deren Familie oder eine besondere histopathologische Diagnose, bspw. eines medullären Schilddrüsenkarzinoms (hohe Wahrscheinlichkeit bzgl. des Vorliegens eines *RET-*assoziierten Tumorrisikosyndroms (TRS)). Auch wenn die humangenetische Beratung der erkrankten Person z. B. wegen fortgeschrittener Erkrankung nicht mehr sinnvoll oder möglich ist, kann dennoch aufgrund der formalgenetischen Risikoerhöhung eine Beratung von Familienmitgliedern indiziert sein.

### 6. Statement: Eine Analyse von Nur-Tumor-Material kann eine erbliche Tumordisposition weder nachweisen noch ausschließen. Dies ist bei der Indikationsstellung zu berücksichtigen

Tumorgewebe beinhaltet somatische Varianten und Keimbahnvarianten. Eine alleinige Tumorsequenzierung kann somit pathogene Keimbahnvarianten – und damit ein erbliches Tumorrisikosyndrom (TRS) – weder sicher nachweisen noch ausschließen [11], [12], [13]. Sequenzierergebnisse, das betroffene Gen, Tumorentität, Histologie und Familienvorgeschichte können allenfalls Hinweise auf ein TRS geben, welches ggf. unter Berücksichtigung des GenDG weiter abgeklärt werden sollte [12].

Eine definitive Unterscheidung allein anhand von Tumorgewebe ist nicht möglich und bedarf daher bei bereits erfolgter Tumoranalyse einer ergänzenden Analyse der Keimbahn-DNA. Daher handelt es sich bei einer ausschließlich im Tumorgewebe nachgewiesenen Genvariante um eine Tumorvariante. Der Begriff somatische Genvariante kann folgerichtig nur dann verwendet werden, wenn eine Keimbahngenese ausgeschlossen wurde. Von den beteiligten Institutionen/Tumorboards sollten Algorithmen etabliert werden, die festlegen, bei welchen klinischen und molekularen Befundkonstellationen die Abklärung einer erblichen Variante empfohlen wird, s. dazu auch Statement 23.

### 7. Statement: Bei Verdacht auf ein erbliches TRS sollte immer eine genetische Untersuchung auf Keimbahnebene angestrebt werden, die alle mit dem TRS assoziierten Gene beinhaltet

**Kommentar:** Bei klinischem Verdacht auf ein erbliches TRS sollte immer eine möglichst umfassende Untersuchung hinsichtlich ursächlicher erblicher Varianten auf Keimbahnebene erfolgen, die alle mit dem TRS assoziierten Gene umfasst, auch wenn in der genetischen Diagnostik aus Tumormaterial bereits eine bestimmte, möglicherweise erbliche Variante detektiert werden konnte. Ist z. B. im Rahmen einer fokussierten Sequenzierung von Tumormaterial bei einer 28-Jährigen mit Mammakarzinom der V. a. eine erbliche TP53-Variante geäußert worden, sollten dennoch in der Keimbahn-Diagnostik (i. d. R. aus Blut) alle mit einer Brustkrebsdisposition assoziierten Gene untersucht werden, nicht nur gezielt die in der Sequenzierung des Tumormaterials beschriebene TP53-Variante. Dies ist sinnvoll, da es sich bei in Tumor-DNA nachgewiesenen pathogenen TP53-Varianten nur sehr selten um konstitutionelle Varianten handelt und deshalb Keimbahn-Varianten in anderen Genen für das frühmanifeste Auftreten des Tumors verantwortlich sein können [12]. Darüber hinaus kann auch eine andere Keimbahnvariante, die z. B. nicht in der genetischen Diagnostik an Tumormaterial identifiziert werden konnte, ursächlich sein. Außerdem wurden vielfach Veränderungen in mehr als einem Tumorrisikogen bei einer Person („doppelte Heterozygotie“) beschrieben [14], [15], [16], [17]. Sowohl für den Patienten selbst als auch dessen Familie kann es fatale Folgen haben, u. a. durch nicht angepasste Früherkennungsempfehlungen, wenn eine mögliche doppelte Heterozygotie nicht identifiziert wird.

### 8. Statement: Die Aussagekraft der tumorgenetischen Diagnostik ist abhängig vom verwendeten Ausgangsmaterial – dies sollte durch entsprechende Angaben im Befundtext nachvollziehbar gemacht werden

**Kommentar:** Aus einer Vielzahl von Gründen (z. B. Alltagstauglichkeit, Möglichkeit einer parallelen, qualitativ hochwertigen histologischen Aufarbeitung) wird heute in der molekularpathologischen Diagnostik an Tumormaterial in aller Regel DNA und RNA aus formalinfixiertem und in Paraffin eingebettetem (FFPE) Gewebe verwendet. Dies gilt insbesondere für alle sogenannten soliden Tumorerkrankungen, da die diagnostische Einordung des Tumortyps und mithin die Therapiesteuerung nach histologischen WHO-Kriterien erfolgt und die Konservierung der Probe mittels FFPE für die Histologie daher eine *conditio sine qua non* darstellt. Diese DNA ist jedoch im Vergleich zu DNA aus unfixierten Zellen in aller Regel von minderer Qualität, unter anderem da die DNA stärker fragmentiert ist und häufiger artifizielle Einzelbasenveränderungen auftreten können [18], [19], [20]. Deshalb ist die Wahrscheinlichkeit, eine medizinisch relevante Variante zu übersehen oder durch Artefakte ein falsch-positives Ergebnis zu erhalten, bei der (alleinigen) Analyse von fixiertem Tumormaterial höher als bei der Analyse von unfixiertem Probenmaterial [21]. Eine Analyse dieses Materials, einschließlich der Analyse komplexer struktureller Varianten, wie bspw. Kopienzahlveränderungen und Genfusionen, ist dennoch erfolgreich möglich, bedarf aber einer besonderen Expertise, die die Bereiche Gewebeaufarbeitung einschließlich Pränanalytik, Sequenzierung, Datenanalyse und Befundinterpretation umfasst. In diesem Zusammenhang sind der Tumorzellgehalt, die Menge und Qualität der Nukleinsäuren [22], die eingesetzte Sequenziermethode, die Qualität der Sequenzierbibliothek, das Paneldesign einschließlich der Verwendung von molekularen Barcodes/Identifiers, die (horizontale) Coverage und (vertikale) Lesetiefe sowie die bioinformatische Auswertung und Plausibilitätsprüfung durch Experten von wesentlicher Bedeutung. Für die Genfusionsanalytik ist eine RNA- oder kombinierte RNA/DNA Sequenzierung einer alleinigen DNA-Sequenzierung vorzuziehen.

Die Sensitivität für die Detektion größerer struktureller Varianten wie einer Deletion ganzer Exons ist abhängig von der Qualität der eingesetzten DNA [23]. Auch aus diesem Grund schließt eine unauffällige Tumorsequenzierung ein erbliches TRS nicht aus, da pathogene strukturelle Varianten unerkannt bleiben können. In einer Vielzahl von Populationen sind strukturelle Varianten als häufiger vorkommende Gründer-Varianten beschrieben worden, z. B. im *BRCA1*-, *BRCA2*-, *CHEK2*- oder dem *APC*-Gen [24]. Für den Nachweis struktureller Keimbahnvarianten ist eine Analyse i. d. R. aus Blut [11], [12], [13] unter Beachtung der Vorgaben des GenDG notwendig.

### 9. Statement: Bei der Untersuchung von Tumor- und nicht-Tumorproben sollte die Herkunft und Qualität der Proben sachgerecht validiert werden

**Kommentar:** Bei Verwendung von DNA aus Zellen in räumlicher Nähe zum Tumorgewebe für die Keimbahnanalytik kann es durch den „field effect“ (Vorkommen von Tumorvarianten in benachbartem, histopathologisch als unauffällig klassifiziertem Gewebe [25]) fälschlicherweise zu einem V. a. ein TRS kommen. Eine Diagnostik hinsichtlich erblicher Varianten an DNA aus Blut-Leukozyten ist auch aufgrund der bereits genannten technischen Limitierungen (siehe Statement 8) gegenüber der genetischen Untersuchung an fixierten Material zu bevorzugen.

Vor der Verwendung von DNA und RNA aus Tumorgewebe solider Tumoren steht die histopathologische Diagnose des Tumors oder die histopathologische Überprüfung der zuvor gestellten Diagnose. Die histopathologische Diagnostik bestimmt die Auswahl des geeigneten tumortragenden FFPE-Blockmaterials oder Frischgewebes, das für die molekularpathologische Untersuchung verwendet wird. Ein Tumorzellgehalt von mindestens 30 %, unter Verwendung von Methoden zur Mikro- und Makrodissektion, ist anzustreben.

### 10. Statement: Die tumorgenetische Diagnostik soll von entsprechenden qualitätssichernden Maßnahmen begleitet werden

**Kommentar:** Bei jedweder tumorgenetischen Diagnostik (Tumor und Blut) sind die entsprechend gültigen Richtlinien z. B. der Bundesärztekammer („RiLiBAeK“) [26] bzw. die entsprechenden Leitlinien und Vorgaben der Fachgesellschaften einzuhalten. Eine Akkreditierung ist anzustreben. Für akkreditierte Einrichtungen gelten die besonderen Vorgaben der DAkkS bzw. der jeweiligen DIN ISO Ziffer (bspw. DIN ISO 15189 und 17020). Die Qualitätssicherung umfasst die gesamte diagnostische Prozesskette einschließlich der Präanalytik und Bioinformatik und eingesetzter Software zur Auswertung von Sequenzierdaten.

### 11. Statement: Bei der tumorgenetischen Diagnostik sollte berücksichtigt werden, dass auch Varianten im Mosaikstatus nachgewiesen werden können, welche auch in anderen, nicht-tumortragenden Geweben, z. B. in den Keimzellen, vorliegen können

**Kommentar:** Postzygotische Mosaike genetischer Varianten, die vor der Geburt entstanden sind, unterliegen im Sinne einer „genetischen Eigenschaft“ dem Anwendungsbereich des GenDG. Solche postzygotischen Mosaike können bei der Analyse des Tumorgewebes detektiert werden, sie können die Keimzellen betreffen und als konstitutionelle Variante an Nachkommen vererbt werden.

Der Verdacht auf das Vorliegen einer Variante in einer solchen Mosaikkonstellation kann sich ergeben:

– im klinischen Kontext
– aufgrund eines geringen Anteils einer Variante (*variant allele fraction*,1Mit der variant allele fraction (auch variant allele frequency, VAF) ist der relative oder absolute Anteil an Sequenz-„reads“ mit der von der Referenzsequenz abweichenden Base in der untersuchten Probe gemeint. Die Variantenallel-Frequenz wird auch als populationsgenetischer Begriff verwendet und gibt an, wie häufig eine Abweichung von der Referenzsequenz in einer bestimmten Population nachweisbar ist. VAF) bei der Analyse von nicht tumortragendem Gewebe
– aufgrund des Vorhandenseins derselben Variante in unterschiedlichen Geweben (beispielsweise syn- oder metachronen Tumoren) einer Person

Die weitere Abklärung erfolgt unter Berücksichtigung des GenDG.

### 12. Statement: Bei Verwendung von Blut oder Speichel als nicht-Tumorprobe sollten mögliche klonale Ereignisse berücksichtigt werden

**Kommentar:** Für die Untersuchung von gesundem, nicht betroffenem Gewebe wird meist DNA aus Leukozyten bzw. aus Speichel verwendet. Durch klonale Hämatopoese können allerdings auch bei nicht erkrankten, meist älteren Personen oder nach Chemotherapie pathogene Varianten in hämatopoetischen Stammzellen entstehen. Solche in geringem Anteil vorkommenden klonalen Varianten gehen mit einem erhöhten Risiko für eine hämatologische Neoplasie, aber auch weiteren nicht-neoplastischen Erkrankungen, insbesondere kardiovaskuläre Erkrankungen, einher (Jaiswal et al., NEJM, 2014 und 2017). Diese „klonale Hämatopoese mit unbestimmtem Potenzial“ (CHIP) [27] kann somit das Vorliegen eines erblichen TRS vortäuschen. Dies wurde bisher u. a. für TP53-Varianten berichtet (fälschliche Diagnose eines Li-Fraumeni-Syndroms) [28].

### 13. Statement: Bei hämatologischen Neoplasien sind bei der Untersuchung auf Keimbahnvarianten besondere Vorkehrungen bei der Auswahl des untersuchten Gewebes zu treffen

**Kommentar:** Viele pathogene Varianten bei hämatologischen Erkrankungen, welche bisher als somatisch angesehen wurden, können erblich sein [29], [30], [31]. Um diese erblichen Varianten von somatischen Varianten zu differenzieren, kann die parallele Analyse von nicht betroffenem Gewebe auch bei hämatologischen Neoplasien notwendig sein. In diesem Rahmen muss darauf geachtet werden, dass die untersuchte Probe nicht mit Leukozyten versetzt ist (was vor allem bei Speichelproben, in geringerem Ausmaß aber auch bei Mundschleimhaut-Abstrichen oder Fingernägeln der Fall sein kann). Um dies zu vermeiden, können zur Abgrenzung erblicher Varianten von somatischen Varianten im Rahmen hämatologischer Neoplasien Nukleinsäuren z. B. aus Haarwurzeln oder Fibroblastenkulturen untersucht werden. Darüber hinaus können hämatopoetische Zellen tumortypische genetische Veränderungen tragen, ohne dass eine hämatologische Systemerkrankung vorliegt (CHIP, s. Statement 12), sodass obligat eine Korrelation mit klinischen, hämatologischen und/oder hämatopathologischen Befunden erfolgen muss.

### 14. Statement: Um therapeutisch relevante Varianten zu erkennen, kann die Liquid Biopsy in definierten klinischen Konstellationen als komplementäre Methode zur gewebebasierten Analytik genutzt werden

**Kommentar:** Die „Liquid Biopsy“ – die Analyse von im Blut (aber auch bspw. in Liquor, Urin, Pleuraerguss, Aszites, Zystenflüssigkeiten) zirkulierender zellfreier DNA (cfDNA) – nimmt bei der Therapiesteuerung von malignen Erkrankungen einen immer größeren Stellenwert ein [32], [33]. Zu diesem Zwecke werden insbesondere zirkulierende Tumorzellen oder Nukleinsäuren untersucht [34]. Klinisch spielt derzeit vor allem die Sequenzierung frei zirkulierender, vom Tumor stammender DNA (ctDNA) aus dem Blut [35], [36], [37] eine Rolle. In definierten klinischen Indikationen kann die Testung auf therapeutisch relevante, insbesondere Resistenz-vermittelnde Varianten genutzt werden. Ferner wird das Monitoring einer onkologischen Erkrankung (bspw. zur Klärung der klinischen Fragestellung eines Rezidivs) in Studien untersucht [38], [39]. Die Liquid Biopsy kann somit die gewebebasierte Analytik ergänzen.

Allerdings ist die gewebebasierte Analytik bei ausreichendem Tumorzellgehalt zur sicheren Erkennung der in der molekular definierten Ersttherapie therapeutisch nutzbaren Varianten, die üblicherweise früh in der Tumorigenese auftreten (Stammvariante, engl. truncal variant), der Liquid Biopsy überlegen und soll daher genutzt werden. Eine Ausnahme besteht, wenn initial nicht ausreichend repräsentatives Gewebe für eine molekulare Analytik verfügbar ist. In diesem Fall kann eine Liquid Biopsy in Erwägung gezogen werden. Bei Verdacht auf eine Resistenzentwicklung unter molekular definierter Ersttherapie soll für eine weitere molekulare Testung zur Identifizierung des Resistenzmechanismus und nachfolgender Zweittherapie, auch wegen der Möglichkeit eines nur histopathologisch nachvollziehbaren sog. Resistenz-vermittelnden Phänotyp-Switches [40], eine gewebebasierte Analytik der klinisch-radiologisch erkannten Rezidivläsion angestrebt werden. Wenn diese aus klinischen Gründen (bspw. schwer erreichbare Tumorläsionen, ECOG-Status des Patienten, Risiko einer Komplikation) nicht möglich ist, kommt die Liquid Biopsy als komplementäres Verfahren in Betracht.

### 15. Statement: Die Liquid Biopsy wird stark durch biologisch-klinische, präanalytische und analytische Faktoren beeinflusst, die kontrolliert und bei der Analyse und Befundinterpretation beachtet werden müssen

**Kommentar:** Zirkulierende, zellfreie DNA (cfDNA) kann auch bei gesunden Probanden, unter physiologischer körperlicher Belastung und bei nicht -onkologischen Erkrankungen im Blut detektiert werden [41], [42], [43]. Die tumor-zugehörige ctDNA stellt häufig nur eine kleine Fraktion der cfDNA dar. Das Vorhandensein von ctDNA bei einer Tumorerkrankung ist stark vom Tumortyp und Tumorstadium abhängig, wobei eine höhere Tumorlast (Stadium IV) prinzipiell mit höheren ctDNA Mengen assoziiert ist [44]: Es gibt jedoch Patienten, die als sogenannte „non- bzw. low-shedder“ bezeichnet werden und bei denen eine ctDNA Analyse nicht möglich ist. Ein negatives Testergebnis schließt daher das Vorhandensein einer klinisch-therapeutisch relevanten Variante in einem Tumor nicht aus. Neben diesen klinisch-biologischen Gesichtspunkten ist ein wesentlicher technischer Aspekt zu beachten: wie auch bei der gewebebasierten Analytik besteht ein direkter Zusammenhang zwischen der Menge der DNA-Moleküle pro Lokus, der Abdeckung und Lesetiefe pro Lokus und der detektierbaren Allelfraktion einer Variante [34]. Die Sensitivität einer Sequenzierung zur Detektion hängt daher auch von der Molekülmenge an der entsprechenden Position ab. Dieser Aspekt spielt bei der Auswahl der geeigneten Methode wie auch bei der Analyse und Interpretation der Ergebnisse eine entscheidende Rolle. Plasma ist gegenüber Serum für die Analyse zu bevorzugen und eine Stabilisierung bzw. Konservierung der cfDNA mittels spezieller Sammelröhrchen ist notwendig [45], [46]. Zu vermeiden ist eine präanalytisch bedingte Dilution der ctDNA-Fraktion durch DNA lysierter Lymphozyten sowie eine Degradation der cfDNA bzw. ctDNA [47]. Temperaturschwankungen und Erschütterungen beim Transport können die cfDNA bzw. ctDNA negativ beeinflussen [48]. Das Einfrieren von Plasmaproben kann nur temporär erfolgen, auch bei -80 Grad Kühlung erfolgt eine Degradation von cfDNA bzw. ctDNA. Diese Aspekte sind bei der Analyse und Befundinterpretation zu beachten.

### 16. Statement: Vor dem Einsatz einer ctDNA-Analyse zur Detektion von Varianten, die als prädiktive Biomarker oder therapeutische Zielstrukturen genutzt werden können, sollte die histopathologische oder hämatologische Diagnose stehen

**Kommentar:** Die Dignitätsfeststellung und Typisierung solider Tumoren gründet auf zyto- und histopathologischen Kriterien, die durch die WHO festgelegt sind. Molekulare Analysen auf DNA- oder RNA-Ebene zur Identifizierung bestimmter Varianten, die in primär histologisch definierten Tumorentitäten gehäuft vorkommen, sind für die Diagnosestellung unterstützend, aber als alleiniges Merkmal im Hinblick auf die Diagnose und ihre Kriterien nicht hinreichend. Daher ist die kontextlose, alleinige Analyse von Varianten, die in ctDNA identifiziert wurden, für primär diagnostische Zwecke, einschließlich der Bestimmung der Dignität und des Tumortyps, nicht geeignet. Studien zur Frage der Früherkennung von Tumoren mittels blutbasierter Tests, einschließlich ethischer Fragestellungen, sind derzeit Gegenstand der Forschung und nicht Bestandteil der etablierten klinischen Versorgung [49].

### 17. Statement: Für eine eindeutige Zuordnung von konstitutionellen und somatischen Varianten bei einer tumorgenetischen Diagnostik von zirkulierender zellfreier DNA („Liquid Biopsy“) ist eine parallele Analyse von nicht-Tumor-DNA erforderlich

**Kommentar:** Im Gegensatz zur DNA aus Tumorgewebe, in dem die Tumorzellen zumeist durch eine Dissektion angereichert werden, ist der Anteil der vom Tumor stammenden DNA-Fragmente in der Zirkulation meist deutlich geringer. Somit wird in einer tumorgenetischen cfDNA-Diagnostik in der Regel zeitgleich prononciert DNA des hämatopoetischen Systems analysiert. D. h. es kann sich bei der cfDNA-Diagnostik nachgewiesen pathogenen Varianten in mit einem TRS-assoziierten Gen um eine pathogene konstitutionelle Variante handeln [50]. Aus diesen Gründen ist eine eindeutige Zuordnung von konstitutionellen und somatischen Varianten im Rahmen einer tumorgenetischen cfDNA-Diagnostik nur durch die parallele Analyse von DNA eines nicht tumortragenden Gewebes/von nicht-Tumorzellen („nicht-Tumor-DNA“) möglich. Insbesondere bei der Diagnostik an cfDNA muss die Möglichkeit einer CHIP (s. entsprechendes Statement 12) in Betracht gezogen werden.

Bezüglich der Anwendung des GenDG muss bei der cfDNA-Diagnostik zwischen einer gezielten, eng umschriebenen Untersuchung (z. B. MRD-Diagnostik) und einer umfangreichen cf-Diagnostik unterschieden werden. Bei letzterer sollte ggf. in Abhängigkeit von der klinischen Konstellation (siehe oben) eine Aufklärung und eine Einwilligung nach GenDG erfolgen.

### 18. Statement: Die Nennung definierter präanalytischer und analytischer Testparameter soll Bestandteil des diagnostischen Befundberichtes sein

**Kommentar:** Der diagnostische Befund soll Parameter beinhalten, die die diagnostische Konstellation einordnen und Rückschlüsse sowohl auf die klinische Wertigkeit der identifizierten Veränderungen als auch über die Aussagekraft der durchgeführten Diagnostik zulässt. Sowohl der Tumortyp als auch der Tumorzellgehalt des untersuchten Tumorareals sollen berichtet werden. Darüber hinaus sollten das verwendete Sequenziergerät, Sequenziertechnologie bzw. -methode, das eingesetzte Genpanel bzw. Kit, Angaben zur Quantität und Qualität der zu analysierenden Nukleinsäuren, Qualität der Sequenzierbibliothek sowie, soweit zutreffend, Art, Typ und Version einer verwendeten Bioinformatikpipeline zur Analyse der Daten genannt werden. Die Beschreibung von genetischen Varianten sollte die Protein- und cDNA-Annotationen, die Variant Allel Fraktion (VAF) und Beurteilung der detektierten Variante (nach humangenetischen oder molekularpathologischen Kriterien, siehe Statements 19 und 22) beinhalten. Der Befund sollte auch Daten (insbesondere Medianwerte) zur vertikalen Coverage (Lesetiefe) und horizontalen Coverage darstellen.

In der humangenetischen Diagnostik, einschließlich hämatologischer Neoplasien, sind die Vorgaben der S2k-Leitlinie „Humangenetische Diagnostik und Beratung“ (medgen 30 (2018) 469-522) sowie der S1-Leitlinie „Molekulargenetische Diagnostik mit Hochdurchsatz-Verfahren der Keimbahn, beispielsweise mit Next-Generation Sequencing“ (medgen 30 (2018) 278-92) zu berücksichtigen.

### 19. Statement: Genetische Varianten sollen nach definierten Nomenklatur-Standards beschrieben werden

**Kommentar:** Um eine Vergleichbarkeit der erhobenen Daten zu gewährleisten sowie Fehlinterpretationen zu vermeiden, sollte die Beschreibung von genetischen Varianten in der tumorgenetischen Diagnostik einer standardisierten Nomenklatur folgen. Nur so kann eine eindeutige Repräsentation von genetischen Daten in einschlägigen klinisch-genetischen Datenbanken wie z. B. ClinVar erreicht werden. Eine Beschreibung von genetischen Varianten sollte somit nach den Empfehlungen der Human Genome Variation Society (HGVS) [51] bzw. dem International Standard for Human Cytogenetic Nomenclature (ISCN) [52] erfolgen. Eine alleinige Benennung von Varianten mit althergebrachten, aber nicht eindeutigen Bezeichnungen, z. B. auf Proteinebene („BRAF V600E“), soll vermieden werden. Ebenso ist für eine eindeutige Variantenbeschreibung neben dem Gen-Namen immer der Bezug zu einem vollständigen (d. h. versionierten) Referenztranskript (z. B. NM...) oder einer Locus Reference Genomic (LRG)-Sequenz notwendig. Für eine nicht transkriptabhängige Variantenbeschreibung sollte möglichst auch eine (zusätzliche) Variantenbeschreibung auf genomischer Ebene erfolgen.

### 20. Statement: Bei paralleler Untersuchung an Tumor- und Normalproben muss sichergestellt werden, dass relevante (potentiell) erbliche Varianten nicht pauschal „maskiert“ werden

**Kommentar:** Gegenüber der alleinigen Testung von DNA aus Tumormaterial bietet die kombinierte Analyse von DNA aus Tumor- und Normalzellen nicht nur eine verbesserte Detektion relevanter somatischer Varianten [53], sondern prinzipiell auch die Möglichkeit, erbliche Varianten zu identifizieren. Werden jedoch zur leichteren Unterscheidung, ob es sich um eine rein somatische Variante handelt, die Variantendaten aus der Normalprobe von denen aus der Tumorprobe subtrahiert („Maskierung“ erblicher Varianten), kann dies zum unbemerkten Ausschluss einer erblichen Variante mit erheblicher klinischer (z. B. therapeutischer) Relevanz führen. Eine solche bioinformatorische Auswertestrategie soll daher nicht angewendet werden. Das durchführende Labor soll im Befund eindeutig nachvollziehbar berichten, welche bioinformatorische Auswertestrategie verfolgt wurde.

### 21. Statement: Die Begriffe „Mutation“ bzw. „Polymorphismus“ sollen nicht zur Beschreibung von Varianten in tumorgenetischen Befunden verwendet werden

**Kommentar:** Eine Abweichung von der Referenzsequenz soll nach den Empfehlungen der HGVS [51] in einem tumorgenetischen Befund nicht mit den Begriffen „Mutation“, „mutiert“ oder „Polymorphismus“ bezeichnet werden. Diese Begriffe sind nicht eindeutig definiert und sollen nicht mehr verwendet werden.

### 22. Statement: In der tumorgenetischen Diagnostik soll die biologische und klinische Bewertung genetischer Varianten mittels eines konsentierten Standards erfolgen

**Kommentar:** Die korrekte Interpretation und Klassifikation genetischer Varianten ist ein mitunter äußerst komplexer Vorgang, der einer umfassenden klinischen und genetisch-biologischen Expertise bedarf. Wie die molekulare und klinische Bewertung einer genetischen Variante zustande kommt, soll transparent und nachvollziehbar sein. Dies ist am besten durch die Verwendung von standardisierten Bewertungsschemata möglich.

Für die Bewertung detektierter somatischer Varianten sind zwei Teilschritte notwendig: i) eine funktionell-biologische Bewertung (Onkogenitätsbewertung) und ii) eine klinische Varianteninterpretation. Für die funktionell-biologische Bewertung, die die funktionellen Implikationen (bspw.: aktivierend, deletär) beschreibt, wird derzeit ein Konsensuspapier des VICC Konsortiums (cancervariants.org) erstellt. Für die Varianteninterpretation im Hinblick auf Ihren therapeutischen Nutzen wurden für die somatische Tumordiagnostik eigene Klassifikationen [54] entwickelt. Diese Klassifikationen ermöglichen die Bewertung der Evidenz für die medikamentös-therapeutische Relevanz einer Variante. Da die Relevanz und der damit verbundene Evidenzgrad sich auf klinische Studien gründen, sind diese einem stetigem Wandel unterworfen. Am National Center for Tumor Diseases (NCT) in Heidelberg wurde 2015 eine Klassifikation entwickelt, um Biomarker hinsichtlich der klinischen Relevanz zu bewerten [55], die sich in der Praxis bewährt hat [56], [57]. In Deutschland wird an vielen Zentren und in Netzwerkstrukturen (bspw. DKTK-MASTER, NNGM, ZPM, DNPM) die Klassifikation des NCT verwendet; daher kann dieses Bewertungsschema zurzeit als Standard in Deutschland angesehen werden. Darüber hinaus existieren zwei weitere große Klassifikationsschemata: Die von der European Society for Medical Oncology (ESMO) entworfenen „ESCAT“-Klassifikation („ESMO scale for clinical actionability of molecular targets“) teilt somatische Tumorvarianten hinsichtlich ihrer klinischen Relevanz in 6 Stufen ein [58]. Die Konsensusempfehlung der AMP, des ACMG, der American Society of Clinical Oncology (ASCO) und des College of American Pathologists (CAP) sieht ebenfalls eine mehrstufige Einteilung vor [59]; ist aber z. B. wegen der spezifischen Ausrichtung auf den FDA-Zulassungsstatus nur bedingt im europäischen Raum einsetzbar und wird derzeit überarbeitet. Eine perspektivische Harmonisierung der verschiedenen Klassifikationsschemata ist notwendig.

Das Bewertungsschema für humangenetisch relevante Sequenzvarianten des American College of Medical Genetics and Genomics (ACMG) und der Association for Molecular Pathology (AMP) [60] gruppiert Varianten in ein fünfstufiges Schema (benigne bzw. wahrscheinlich benigne Variante, Variante unklarer Signifikanz, wahrscheinlich pathogene bzw. pathogene Variante). Dieses Schema wird kontinuierlich weiterentwickelt und aktualisiert und wurde ausdrücklich für die Bewertung von hoch penetranten, erblichen Varianten entworfen. Es eignet sich daher nur bedingt für die Interpretation von somatischen Varianten in der Tumordiagnostik. Das Schema ist jedoch speziell für die therapeutische Bewertung (Ansprechen auf sog. PARP-Inhibitoren) von Varianten in Genen, die für die homologe Rekombinationsreparatur (HRR) kodieren, mit gering modifizierter Nomenklatur (Verwendung des Begriffs deletär statt pathogen) adaptiert worden und schließt dort auch somatische Varianten ein (bspw. PROFOUND-Studie [61]). Ferner ist zu beachten, dass zunehmend genspezifische Adaptionen des ACMG/AMP-Regelwerks publiziert werden, die auf die genspezifischen Besonderheiten bei der Variantenbewertung gezielt eingehen (z. B. [62], [63]).

Im Befundbericht soll dokumentiert sein, welches Bewertungsschema verwendet wurde und welche Kriterien des jeweiligen Bewertungsschemas zur entsprechenden Klassifizierung geführt haben. Die evidenzbasierte Bewertungsgrundlage für die jeweilige Variantenklassifikation sollte mitdokumentiert werden.

### 23. Statement: Bei allen umfangreichen tumorgenetischen Untersuchungen an Tumormaterial sollten potentiell erbliche Varianten, die mit einem TRS assoziiert sein könnten, berichtet werden

**Kommentar:** Wird eine genetische Analyse ausschließlich an Tumor-DNA durchgeführt, ist eine Identifikation oder ein Ausschluss von erblichen Varianten nicht möglich [21].

Die ESMO empfiehlt, eine Auswertung auf mögliche erbliche, mit einem TRS assoziierte Veränderungen – so der Patient über eine solche Untersuchung aufgeklärt wurde und damit einverstanden ist – bei allen umfangreichen tumorgenetischen Untersuchungen routinemäßig durchzuführen [12]. Als (wahrscheinlich) pathogen klassifizierte Varianten in sogenannten „High Actionability Cancer Susceptibility Genes“ sollen demnach unter Berücksichtigung der Tumorerkrankung, der Varianten-Allelfraktion und des Erkrankungsalters ggf. als potentiell erbliche Varianten berichtet werden [12]. Eine Keimbahntestung in geeignetem Material kann dann erfolgen. Die Empfehlungen beziehen sich auf häufige Tumorerkrankungen. Hierbei handelt es sich um einen Kompromiss, um den Aufwand einer Auswertung auf ein TRS auch bei hoher Probenzahl durchführen zu können.

Alle Institutionen, die umfangreiche tumorgenetische Untersuchungen durchführen, sollten über eine schriftlich niedergelegte Verfahrensanweisung verfügen, in dem das entsprechende Vorgehen dargelegt wird. Eine Harmonisierung, z. B. durch Empfehlungen von nationalen und internationalen Fachgesellschaften, ist anzustreben.

### 24. Statement: Die Ergebnisse von Variantenbewertungen sollten in öffentlichen Datenbanken abgelegt werden

**Kommentar:** Für die Variantenbeurteilung in der tumorgenetischen Diagnostik ist ein übergreifender, populationsbezogener Vergleich mit der „Normalpopulation“ essentiell für die Einordnung einer seltenen Variante. Daher sollten die Varianten – unter Beachtung (inter-)nationaler (z. B. DSGVO) und lokaler Datenschutzregelungen und eindeutig geregelter Zugriffsrechte – in öffentlichen Datenbanken gespeichert werden. Im Umkehrschluss sollte daher jedes Labor, das auf die öffentlich gespeicherten Daten zugreifen möchte, die selbst generierten Daten vollständig in den entsprechenden öffentlichen Datenbanken (z. B. ClinVar-Datenbank) ablegen.

### 25. Statement: Bei umfangreichen tumorgenetischen Untersuchungen ist eine interdisziplinäre Bewertung der Ergebnisse anzustreben

**Kommentar:** Die Beurteilung komplexer Sequenzdaten mit einem unter Umständen hohen Anteil von potentiell medizinisch relevanten Varianten erfordert eine umfassende Fachexpertise, die den gesamten diagnostischen Prozess von der Präanalytik über die Sequenzierung bis hin zur bioinformatischen Analyse, Befunderstellung und Diskussion in einem (molekularen) Tumorboard umfasst. Dies schließt auch den Umgang mit Datenbanken und biomathematischen Prädiktionsmodellen ein. Die klinische Interpretation von konstitutionellen und somatischen Varianten sollte je nach klinischer Konstellation in enger Zusammenarbeit zwischen Onkologen, Pathologen, Naturwissenschaftlern, Bioinformatikern, Humangenetikern und anderen Fachvertretern (entsprechend der Tumorboards) interdisziplinär erfolgen. Der Einschluss in prospektive klinische Studien ist anzustreben.

## Appendix

**Kategorie:** S1-Leitlinie

**AWMF-Reg. Nr.:** 078-017

**Interessenkonflikte:** Die Erklärung zu potenziellen Interessenkonflikten wurde nach den Kriterien des AWMF-Formblattes eingeholt. Bei dieser Leitlinie hat keiner der beteiligten Experten oder Autoren einen Interessenskonflikt, insofern gab es auch keine Enthaltungen bei der Bewertung der Leitlinie. Die Angaben zu den Interessenkonflikten wurden von Dr. med. Bernd Auber geprüft und freigegeben.

**Verabschiedung der S1-Leitlinie durch die Vorstände der folgenden Gesellschaften und Verbände:**

Arbeitskreis erbliche Tumorerkrankungen (AET) der Deutschen Krebsgesellschaft (DKG)

Berufsverband Deutscher Humangenetiker (BVDH)

Bundesverband Deutscher Pathologen (BDP)

Deutsche Gesellschaft für Gynäkologie und Geburtshilfe e. V. (DGGG)

Deutsche Gesellschaft für Hämatologie und Onkologie (DGHO)

Deutsche Gesellschaft für Humangenetik (GfH)

Deutsche Gesellschaft für Klinische Chemie (DGKL)

Deutsche Gesellschaft für Neuropathologie und Neuroanatomie (DGNN)

Gesellschaft für Pädiatrische Onkologie und Hämatologie (GPOH)

Deutsche Gesellschaft für Pathologie (DGP)

**Fertigstellungsdatum:**

12/2021

**Nächste Überprüfung geplant:**

06/2023

**Ansprechpartner**

Dr. med. Bernd Auber, MBA

Institut für Humangenetik

Medizinische Hochschule Hannover

Carl-Neuberg-Str. 1

30625 Hannover

Tel.: +49 511 532 8719

Fax: +49 511 532 5865

E-Mail: auber.bernd@mh-hannover.de

Prof. Dr. med. Albrecht Stenzinger

Institut für Pathologie

Universitätsklinikum Heidelberg

Tel.: +49 6221 56-34380

Fax: +49 6221 56-5251

E-Mail: Albrecht.Stenzinger@med.uni-heidelberg.de

**Leitlinienkoordination:**

Dr. med. Bernd Auber, MBA, Hannover

Prof. Dr. med. Albrecht Stenzinger, Heidelberg
